# Comparative Analysis of the Antineoplastic Activity of C_60_ Fullerene with 5-Fluorouracil and Pyrrole Derivative In Vivo

**DOI:** 10.1186/s11671-016-1775-0

**Published:** 2017-01-05

**Authors:** O. V. Lynchak, Yu I. Prylutskyy, V. K. Rybalchenko, O. A. Kyzyma, D. Soloviov, V. V. Kostjukov, M. P. Evstigneev, U. Ritter, P. Scharff

**Affiliations:** 1 Taras Shevchenko National University of Kyiv, 64 Volodymyrska Str, 01601 Kyiv, Ukraine; 2 Joint Institute for Nuclear Research, Dubna, Moscow Region Russia; 3 Moscow Institute of Physics and Technology, Dolgoprudny, Moscow Region Russia; 4 Sevastopol State University, Sevastopol, Crimea Ukraine; 5 Belgorod State University, 85 Pobedy Str, 308015 Belgorod, Russia; 6 Institute of Chemistry and Biotechnology, Technical University of Ilmenau, 25 Weimarer Str, 98693 Ilmenau, Germany

**Keywords:** C_60_ fullerene, 5-Fluorouracil, Pyrrole derivative, Colorectal cancer, Small-angle X-ray scattering, Molecular modeling

## Abstract

The antitumor activity of pristine C_60_ fullerene aqueous solution (C_60_FAS) compared to 5-fluorouracil (5-FU) and pyrrole derivative 1-(4-Cl-benzyl)-3-Cl-4-(CF_3_-fenylamino)-1H-pyrrol-2.5-dione (MI-1) cytostatic drugs was investigated and analyzed in detail using the model of colorectal cancer induced by 1.2-dimethylhydrazine (DMH) in rats. The number, size, and location of the tumors were measured, and the pathology was examined. It was found that the number of tumors and total lesion area decreased significantly under the action of C_60_FAS and MI-1. Because these drugs have different mechanisms of action, their simultaneous administration can potentially increase the effectiveness and significantly reduce the side effects of antitumor therapy.

## Background

C_60_ fullerene has been intensively investigated in the last decades mainly because of their vast range of potential applications in nanotechnology. The nearly spherical C_60_ molecule (diameter is 0.72 nm) can currently be routinely synthesized and is characterized by high chemical stability and biological activity in vitro and in vivo [1–4]. C_60_ fullerenes are soluble in nonpolar organic solvents [5] and can be transferred into the water by means of special procedures [6–8]. These properties enable fullerene to utilize in biological objects due to their ability to easily penetrate the cell lipid membrane [9–12]. At low (near-physiological) concentrations, C_60_ fullerenes demonstrate no acute toxic effect in normal cells [13–17]. It was also reported that C_60_ fullerene can be used in antitumor therapy [18, 19], including the photodynamic therapy for the treatment of oncological diseases [20–23] as well as the targeted delivery of traditional drugs into cancer cells [24, 25].

Colon cancer (colorectal cancer) is one of the most common diagnoses in the world, which corresponds to approximately 1.4 million patients in 2012, and this number is foreseen to increase by almost half in 2035 [26, 27]. The 5-year relative survival rate for patients with colorectal cancer is in between 12 and 90% and strongly depends on the time passed till diagnosis, the stage of the disease, and the conducted treatment [28].

Colorectal carcinogenesis is a heterogeneous and complex multistage process, which involves violations of a homeostatic control of proliferation, differentiation, and apoptosis of intestine epithelial cells. The causes of these disorders are genetic mutations of transforming oncogenes and deletions or mutations of DNA repair genes and tumor suppressor genes. In addition, indirectly acting factors, such as diet and environmental factors, may contribute to the development of cancer via the modulation of signaling pathways of intestinal epithelial cells. Multiple mutations over many years are required for the occurrence of abnormal growth that leads to colon cancer [29, 30].

Artificially induced (by a specific carcinogen) tumors in laboratory animals provide an opportunity to investigate various aspects of carcinogenesis that cannot be effectively studied in the human body directly [31–33]. Therefore, a significant number of the experimental models of tumorigenesis in various organs were developed. The dimethylhydrazine model is an effective tool to study the features of intestinal carcinogenesis under the action of therapeutic agents. Morphological changes in rat intestine caused by the 1.2-dimethylhydrazine (DMH) are similar to those that occur during the colon cancer development in human tissues [32, 34].

The aim of this work was to study and analyze the antitumor activity of pristine C_60_ fullerene aqueous solution (C_60_FAS) compared with the effect of pyrrole derivative 1-(4-Cl-benzyl)-3-Cl-4-(CF_3_-fenylamino)-1H-pyrrol-2.5-dione (MI-1) and the most commonly used 5-fluorouracil (5-FU) drug on the DMH-induced colon cancer model in rats.

## Methods

A highly stable reproducible C_60_FAS in concentration 0.15 mg/ml was prepared according to the protocol [7, 8].

Small-angle X-ray scattering (SAXS) experiments were carried out on instrument with high-intensity microfocus rotating Cu anode X-ray generator in the laboratory for advanced studies of membrane proteins (Moscow Institute of Physics and Technology, Dolgoprudniy, Russia), using a standard transmission configuration. An X-ray wavelength of *λ* = 1.54 Å was used, resulting a momentum transfer *Q* in the range of 0.007–0.2 Å^−1^, where *Q* = (4π/*λ*) × sin(*θ*/2) and *θ* is the scattering angle. The samples studied were placed in borosilicate capillaries of 1.5-mm diameter and 0.01-mm wall thickness (W. Muller, Berlin, Germany). Water was used as a buffer sample. Center of beam line and conversation channel to value of module q-vector was done using silver behenate.

Pyrrole derivative 1-(4-Cl-benzyl)-3-Cl-4-(CF_3_-fenylamino)-1H-pyrrole-2.5-dione (MI-1) was synthesized by the ChemBioCenter in Taras Shevchenko National University of Kyiv [35, 36].

2.4-Dihydroxy-5-fluoropyrimidine (5-FU; “Darnitsa,” Ukraine) was used in experiments.

The study was conducted on 60 white male rats of the “Wistar” line weighing 180–200 g. The animals were kept under standard conditions in the vivarium of the ESC “Institute of Biology and Medicine,” Taras Shevchenko National University of Kyiv. Animals had free access to food and water. All experiments were conducted in accordance with the international principles of the European Convention for the protection of vertebrate animals under a control of the Bio-Ethics Committee of the abovementioned institution.

Colorectal cancer was induced by DMH (Sigma-Aldrich, USA), which was administered weekly for 20 weeks at a dose of 20 mg/kg (the dose and duration of administration are sufficient for the induction and subsequent development of colorectal cancer in rats) [37]. Starting from the 21 weeks of the experiment, the animals were divided into four groups: I—control (animals were given subcutaneous injections of saline weekly); II—animals were treated by subcutaneous injections of C_60_FAS at a dose of 2.0 mg/kg weekly; III—animals were treated by subcutaneous injections of 5-FU at a dose of 45 mg/kg weekly; IV—animals were given MI-1 intragastric at a dose of 2.7 mg/kg daily. After 27 weeks, the animals were submitted to euthanasia with CO_2_. Immediately after euthanasia, the intestine was removed (from the cecum to the anus) and was opened with scissors in the antimesenteric border and stretched in Styrofoam plates for cleaning with 0.9% NaCl. The number, location, and area of the neoformations were determined by special procedures [38]. The tumors were excised and fixed in 10% neutral buffered formalin and then subjected to standard histological processing and staining with hematoxylin and eosin. Slides were analyzed using a light microscope Olympus BX-41 (Germany). The obtained results were treated by conventional methods of statistics using StatPlus2009 software.

Modeling of the coordination complex of C_60_ fullerene with Fe ion was accomplished using the C_60_(OH)_24_ fullerenol model of the C_60_ molecule. The initial structure of C_60_(OH)_24_Fe(H_2_O)_3_ was built by creating its geometry similar to the well-established geometry of the Fe(H_2_O)_3_ octahedral cluster in water [39]. The spatial structure of C_60_(OH)_24_Fe(H_2_O)_3_ was preliminary optimized by means of the MM+ molecular mechanics method in HyperChem 8.0. The final optimization was made by means of the PM6 method in Gaussian09W.

## Results and Discussion

Since the C_60_ fullerene particles size directly correlates with their biodistribution and toxicity [3, 15, 16], SAXS study of C_60_FAS was performed.

**Fig. 1:**
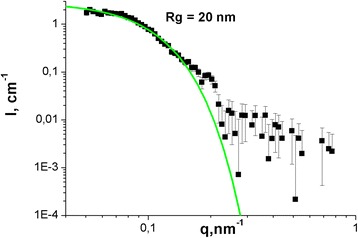
Experimental SAXS curve for C_60_FAS with concentration 0.15 mg/ml. The *solid line* illustrates the calculated scattering

The experimental SAXS curve of C_60_FAS is shown in Fig. 1. The absence of specific peculiarities on the curve suggests that the particles are polydisperse in size. It is in a good agreement with probe microscopy data [40, 41]. At sufficiently small *q* values (*qR*_g_ < 1), the scattered intensity can be described by the Guinier approximation

$$ I(q)=I(0)\cdotp \exp \left\{-{\left(q{R}_{\mathrm{g}}\right)}^{2/3}\right\} $$

with two parameters, which are the forward scattered intensity, *I*(0), and the apparent radius of gyration, *R*_g_. The found gyration radius of clusters, *R*_g_, is about 20 nm. For spherical homogeneous approximation in the case of C_60_FAS [6, 40, 42, 43], the C_60_ fullerene nanoparticle size can be estimated as 2 × (5/3)^1/2^ × *R*_g_, which gives the effective diameter about 52 nm.

All experimental rats had tumors in the colon. The vast majority of tumors were detected in the descending colon, which is typical for this model of carcinogenesis [32]. Neoplasms had different sizes and shapes (flat, protruding, raised, recessed, granular, and agranular) with exophytic and entophytic types of growth. From the histological studies, the adenoma and adenocarcinoma were found (data not shown). Rats treated by C_60_FAS mainly had adenoma.

Table 1 shows the average size of tumors, the average number of tumors, and the total area of lesions of different parts of the colon under the action of DMH.

**Table 1:** The effectiveness of 5-FU, MI-1, and C_60_FAS at DMH-induced colon cancer (*M* ± *m*; *n* = 15)

	Parts of the large intestine	Experimental group			
		DMH	DMH + 5-FU	DMH + C_60_FAS	DMH + MI-1
Average number of tumors, per rat	Cecum	2.8 ± 0.4	1.8 ± 0.3	1.2 ± 0.1*	1.3 ± 0.2*
	Colon	11.5 ± 1.0	9.0 ± 0.4	8.3 ± 0.2*	8.6 ± 0.7*
	Rectum	2.6 ± 0.3	2.8 ± 0.4	2.0 ± 0.3	1.9 ± 0.2
	All parts	16.8 ± 1.0	13.5 ± 0.7	11.5 ± 0.3*	11.8 ± 0.8*
Average area of tumors, mm^2^	Cecum	9.6 ± 1.8	6.4 ± 1.0	9.4 ± 1.7	5.0 ± 1.0*
	Colon	15.0 ± 1.6	9.0 ± 0.5	11.2 ± 0.7	11.6 ± 0.5
	Rectum	22.0 ± 2.1	18.6 ± 2.1	18.4 ± 1.1	16.1 ± 2.0
	All parts	14.5 ± 1.1	10.3 ± 0.5*	12.1 ± 0.6	11.4 ± 0.5*
Total area of tumors, per rat, mm^2^	Cecum	30.5 ± 8.1	11.1 ± 2.0	10.5 ± 1.6*	6.3 ± 1.2*
	Colon	158.9 ± 11.7	79.7 ± 1.8*	95.0 ± 7.8*	100.9 ± 10.3*
	Rectum	51.9 ± 5.1	45.8 ± 5.5	34.6 ± 3.3*	29.2 ± 4.7*
	All parts	241.3 ± 16.4	136.6 ± 6.1*	140.0 ± 10.3*	136.4 ± 12.9*

*p < 0.05 relative to control

Introduction of C_60_FAS caused the reduction of the number of tumors in the cecum by 57% and the total lesion area by 65% (Table 1). Under the 5-FU action, only the downward trend of these rates was observed.

In the colon, the number of tumors has reduced by 28% and the total lesions area by 40% under the C_60_FAS action; it was close to the 5-FU effects (Table 1). A tendency to the reduction of the average area of tumors was also observed.

In the rectum, the number of tumors had demonstrated a tendency to reduction and the total lesions area had decreased by 33% under the C_60_FAS action (Table 1).

Under the action of traditional anticancer drug, 5-FU, only a tendency to decrease the average tumor area and the total lesion area was noted.

In total, in the colon, C_60_FAS reduced the number of tumors by 31% and the total lesion area by 42% (Table 1). Nearly the same quantitative changes had also occurred under the action of 5-FU, viz. the tumor area decreased by 28% and the total lesion area by 43%.

The antitumor effect of MI-1 was similar to C_60_FAS and 5-FU: the average number of tumors, tumor size, and total lesions area were reduced (Table 1). Note that MI-1 is characterized by low toxicity in the gastrointestinal tract and hematopoietic organs [44, 45].

Thus, therapeutic administration of C_60_FAS, 5-FU, and MI-1 leads to a reduction of the number of DMH-induced tumors and colon lesion area in rats. MI-1 and 5-FU drugs (the latter is related to antimetabolites and pyrimidine antagonists, whose antitumor effect manifests itself as a result of 5-fluorouracil converted to fluorodeoxyuridine monophosphate, fluorodeoxyuridine triphosphate, and fluorouridine triphosphate [46]) violate DNA synthesis and, as a consequence, inhibit cell division. The mechanism of C_60_ fullerene action is different and can be related to its antioxidant effect [47, 48] that causes the epigenetic changes in tumor cells, which inhibit their further growth. As a result of the DMH metabolism in the liver, the formation of electrophilic diazonium ions occurs [32], which causes an oxidative stress. C_60_ fullerene, being a powerful antioxidant, is able to prevent the progression of tumors at an early stage. Based on the fact that MI-1 and C_60_ fullerene have different mechanisms of action, the observed effectiveness of their combined action on the colorectal carcinogenesis model can therefore be explained, at least in part. Hence, the simultaneous administration of MI-1 and C_60_ fullerene can potentially increase the effectiveness of anticancer therapy and reduce side effects, reported recently for other C_60_ fullerene-drug combinations [49, 50]. Let us further discuss the possible mechanism by which the antioxidant effect of C_60_FAS may operate in the system investigated in the present work.

It is currently considered that the water-soluble C_60_ fullerene derivative, C_60_(OH)_24_ fullerenol, exerts its protective role against doxorubicin-induced toxicity due to the removal of free iron, forming a stable “fullerenol-iron” complex [51]. Because the cancer cells grow rapidly in response to iron, the formation of colorectal cancer can therefore be inhibited by the removal of free iron in the form of “fullerenol-iron” nanoparticles. Let us further consider the possibility of formation of stable complex between pristine C_60_ fullerene used in the present work (not containing the –OH groups), and the iron ions, which cannot be expected from the very beginning.

**Fig. 2:**
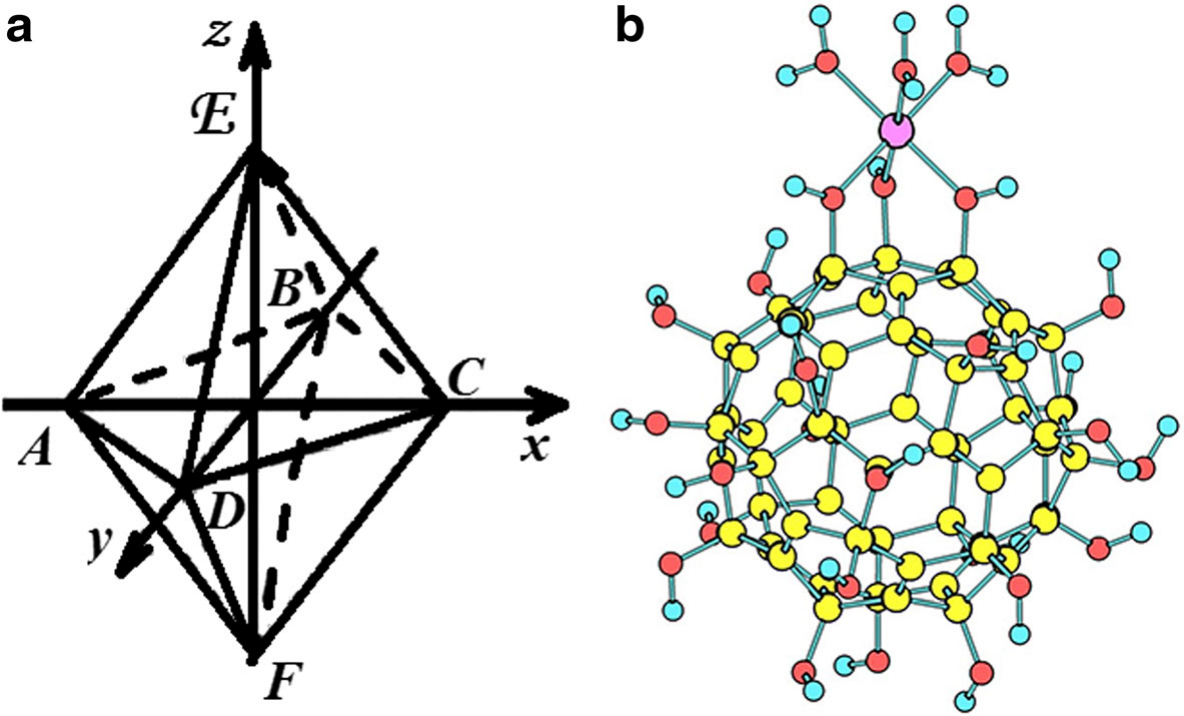
Hydrated iron ion (Fe ion is located in the center of octahedral. Water molecules are located in heights (**a**); the calculated structure of the “C_60_ fullerene-iron” complex (**b**)

It has long been established that the dissolution of pristine C_60_ fullerene in water is due to the formation of solvation shell around the C_60_ molecule tightly attached to it [5, 6, 43, 52, 53]. However, recent data have evidenced that the molecular dissolution of the C_60_ fullerene in water, at least in part, is promoted by the attachment of a certain number of OH groups to the C_60_ fullerene surface, i.e., the pristine C_60_ fullerene in water becomes partially hydroxylated [7, 54]. The number of the –OH groups attached to the surface is still unknown, but the literature data suggest that the C_60_ molecule may incorporate 2 to 44 hydroxyls freely migrating around the C_60_ fullerene surface [55]. On the other hand, the coordination number of the transition metal ions, including the iron ions such as Fe^+2^ and Fe^+3^, typically equals to 6. In water, the Fe ions are coordinated by six water molecules forming regular octahedral. Taking this into account, it can be assumed that the “C_60_ fullerene-iron” complex may be formed by the coordination of three –OH groups on the surface of C_60_ fullerene and three water molecules from the bulk solution. The corresponding energy-minimized structure of the proposed complex is given in Fig. 2a. The stability of such structure is verified by the fact that the resultant geometry of the coordinated cluster well matches the geometry of the stable Fe octahedral in water (Fig. 2b), i.e., the distance between Fe ion and water oxygen in hydrated ions is close to the same distance in C_60_ fullerene-iron octahedral (≈2 Å). We consider that such complexation of Fe to water-soluble pristine C_60_ fullerene may explain the antioxi-dant effect of C_60_FAS synergistically/additively operat-ing together with doxorubicin [49] or MI-1 in suppres-sion the tumor growth.

## Conclusions

It was found that the administration of pristine C_60_ fullerene aqueous solution (C_60_FAS), 5-fluorouracil (5-FU), and pirrole derivative 1-(4-Cl-benzyl)-3-Cl-4-(CF3-fenylamino)-1H-pyrrol-2.5-dione (MI-1) reduces the number of 1.2-dimethylhydrazine-induced tumors and colon lesion area in rats. The antitumor effects of C_60_FAS and MI-1 demonstrated no significant difference but additively operate in tumor suppression on their simultaneous use. These results point out the possibility of the practical application of C_60_FAS and MI-1 in the combination therapy of colorectal cancer.

## Abbreviations

5-FU: 5-Fluorouracil
DMH: 1,2-Dimethylhydrazine
MI-1: Pyrrole derivative 1-(4-Cl-benzyl)-3-Cl-4-(CF_3_-fenylamino)-1H-pyrrol-2,5-dione
C_60_FAS: C_60_ fullerene aqueous solution
